# Assessment of severe malaria in a multicenter, phase III, RTS, S/AS01 malaria candidate vaccine trial: case definition, standardization of data collection and patient care

**DOI:** 10.1186/1475-2875-10-221

**Published:** 2011-08-04

**Authors:** Johan Vekemans, William Kabore, Solange Soulanoudjingar, Portia Kamthunzi, Nahya Salim, Walter Otieno, Samwel Gesase, David Schellenberg, the Clinical Trials Partnership Committee

**Affiliations:** 1 GlaxoSmithKline Biologicals, Wavre, Belgium; 2 KEMRI-Wellcome Trust Research Programme, Kisumu, Kenya; 3 London School of Hygiene and Tropical Medicine, University of London, London, UK; 4 Institut de Recherche en Sciences de la Santé, Centre Muraz, Burkina Faso; 5 Albert Schweitzer Hospital, Kumasi, Gabon; 6 Kintampo Health Research Centre, Ghana Health Service, Kintampo, Ghana; 7 School of Medical Sciences, Kwame Nkrumah University of Science and Technology, Kumasi, Ghana; 8 Kumasi Centre for Collaborative Research, Kumasi, Ghana; 9 Centro de Investigação em Saúde de Manhiça, Manhiça, Mozambique; 10 University of North Carolina (UNC) Project, Lilongwe, Malawi; 11 Ifakara Health Institute, Ifakara, Tanzania; 12 KEMRI/CDC Research and Public Health Collaboration, Kisumu, Kenya; 13 National Institute of Medical Research, Dar es salaam, Tanzania; 14 PATH Malaria Vaccine Initiative, Washington, USA

## Abstract

**Background:**

An effective malaria vaccine, deployed in conjunction with other malaria interventions, is likely to substantially reduce the malaria burden. Efficacy against severe malaria will be a key driver for decisions on implementation. An initial study of an RTS, S vaccine candidate showed promising efficacy against severe malaria in children in Mozambique. Further evidence of its protective efficacy will be gained in a pivotal, multi-centre, phase III study. This paper describes the case definitions of severe malaria used in this study and the programme for standardized assessment of severe malaria according to the case definition.

**Methods:**

Case definitions of severe malaria were developed from a literature review and a consensus meeting of expert consultants and the RTS, S Clinical Trial Partnership Committee, in collaboration with the World Health Organization and the Malaria Clinical Trials Alliance. The same groups, with input from an Independent Data Monitoring Committee, developed and implemented a programme for standardized data collection.

The case definitions developed reflect the typical presentations of severe malaria in African hospitals. Markers of disease severity were chosen on the basis of their association with poor outcome, occurrence in a significant proportion of cases and on an ability to standardize their measurement across research centres. For the primary case definition, one or more clinical and/or laboratory markers of disease severity have to be present, four major co-morbidities (pneumonia, meningitis, bacteraemia or gastroenteritis with severe dehydration) are excluded, and a *Plasmodium falciparum *parasite density threshold is introduced, in order to maximize the specificity of the case definition. Secondary case definitions allow inclusion of co-morbidities and/or allow for the presence of parasitaemia at any density. The programmatic implementation of standardized case assessment included a clinical algorithm for evaluating seriously sick children, improvements to care delivery and a robust training and evaluation programme for clinicians.

**Conclusions:**

The case definition developed for the pivotal phase III RTS, S vaccine study is consistent with WHO recommendations, is locally applicable and appropriately balances sensitivity and specificity in the diagnosis of severe malaria. Processes set up to standardize severe malaria data collection will allow robust assessment of the efficacy of the RTS, S vaccine against severe malaria, strengthen local capacity and benefit patient care for subjects in the trial.

**Trial registration:**

Clinicaltrials.gov NCT00866619

## Background

Malaria remains a considerable burden despite progress in implementation of malaria control policies. It is estimated that malaria causes 709,000 deaths every year in Africa; 85% in children less than five years of age [1]. Used in conjunction with existing interventions such as indoor residual insecticidal spraying, long-lasting insecticide-impregnated bed nets, intermittent preventive anti-malarial therapy in pregnancy, and prompt treatment with an effective anti-malarial, an effective malaria vaccine for young children in Africa has the potential to reduce the burden of the disease substantially.

The malaria vaccine at the most advanced stage of development is RTS, S, a pre-erythrocytic vaccine candidate containing *Plasmodium falciparum *circumsporozoite surface antigen. This vaccine is formulated with new proprietary Adjuvant Systems - AS01 or AS02 [2]. Earlier studies have demonstrated the potential of RTS, S/AS02 [3,4] and RTS, S/AS01 to provide partial protection against uncomplicated malaria [5]. In addition to prevention of uncomplicated malaria, one of the main aims of malaria vaccines is to prevent severe malaria. Although severe malaria can present in many different ways, typical symptoms in young children include severe anaemia, respiratory distress, hypoglycaemia, repeated seizures, acidosis, prostration and fever [6]. Severe malaria is most commonly caused by *P. falciparum*, and in areas of stable endemicity is mainly a disease of children under five years of age [6].

Efficacy against severe malaria is a key factor that will determine evidence-based decisions on the implementation of new malaria control tools [7,8]. Evidence from phase II RTS, S trials showed the vaccine can provide protection against severe disease. In the proof-of-concept study conducted in Mozambique, the RTS, S/AS02 vaccine prevented 49% (95% CI 12-71) of severe malaria episodes during 18 months of follow-up [4]. In a study in Kenya and Tanzania, out of 809 children, half of whom had received RTS, S/AS01, one case of severe malaria was reported in the experimental malaria vaccine group as compared with eight in the control group, over an eight months follow-up period [5]. These encouraging results need confirmation in a larger phase III multicentre study with a robust and standardized assessment of cases of severe malaria.

Previous multi-centre studies on severe malaria have demonstrated that standardized assessment of cases is challenging [9]. Success depends on the use of common case definitions, standardized investigation of patients and the quality of diagnosis and care. Accurate diagnosis of severe malaria can be difficult because of the diversity in presentation and because its symptoms overlap with those of many common febrile childhood illnesses including pneumonia, meningitis and sepsis [10-12]. For example, an autopsy study of 31 children diagnosed with cerebral malaria in Malawi showed that 23% had died from other causes [13].

The current, internationally accepted definition of severe malaria in children is based on the need for prompt treatment of children with a medical history and clinical presentation compatible with severe malaria, given the high complication and fatality rate of this condition [6,14]. In clinical practice, the threshold for initiation of anti-malaria treatment needs to be low, and is sometimes based on syndromic assessment only rather than on investigation-supported diagnosis. In these circumstances, a highly sensitive but non-specific case definition is appropriate to determine treatment. However, use of a sensitive but non-specific case definition as a trial endpoint would be expected to result in an underestimation of vaccine efficacy [15]. Conversely, by using a highly specific but poorly sensitive case definition, a large sample size will be required to provide a sufficient number of cases. An ideal case definition for the purpose of a clinical trial thus needs to combine a level of specificity that provides an accurate estimate of vaccine efficacy with a level of sensitivity that optimizes the power of the study [16,17]. A World Health Organization (WHO) expert study group has developed recommendations on appropriate criteria for severe malaria case definitions for use as a vaccine trial endpoint [8].

The results of phase II studies led to a decision to employ the RTS, S/AS01 vaccine formulation for the pivotal phase III trial. The objectives and design of the phase III RTS, S/AS01 study are described in a companion paper [18]. This paper provides a detailed description of the case definitions of severe malaria used for the trial, the work undertaken to ensure standardized assessment of severe malaria across different study centers and the efforts made to ensure optimal patient care.

## Severe malaria endpoint case definitions for evaluation in the RTS, S/AS01 Phase III trial

The case definitions of severe malaria were developed prospectively through a literature review and consensus meeting of the Clinical Trial Partnership Committee (CTPC) severe malaria working group and expert consultants, in collaboration with representatives of the WHO and the Malaria Clinical Trials Alliance (MCTA). The CTPC includes representatives of all the research centres collaborating on the RTS, S clinical development programme, GlaxoSmithKline and the Malaria Vaccine Initiative.

The main objective of this discussion was the development of a primary case definition which balanced sensitivity and specificity, reflecting true paediatric malaria cases at risk of an adverse outcome, usually needing hospital admission, taking into account the fact that symptoms often overlap with other diseases, and that other co-morbidities can be present.

A retrospective study of 1,361 children admitted to a Kenyan District hospital and 4,583 community controls showed how the specificity of a case definition for severe malaria varied depending upon the intensity of transmission, the parasite density threshold applied, the markers of severity that were included and upon how co-morbidities were dealt with during analysis. Among children under two years old who presented with symptoms compatible with severe malaria, logistic regression models showed that the malaria-attributable fraction of these cases was only 85%. This increased to 89% when children with identified co-morbidities (similar to those proposed here) were excluded and to 95% when a threshold of 2500 parasites/μL was also applied [14].

Table 1 illustrates the primary and secondary case definitions of severe malaria that will be used in the analysis of clinical data from the phase III RTS, S/AS01 clinical trial. The primary case definition of severe malaria will include a parasite density threshold of >5,000 parasites/μL, and one or more markers of disease severity. Children with major co-morbidities will be excluded from the primary case definition. As described in a companion paper [18], the parasite density threshold selected for clinical malaria (5,000 parasites per μl) is expected to provide a minimum specificity of 80% for all age groups and transmission settings. The same threshold will be used in the definition of severe malaria. Secondary case definitions with increased sensitivity will also be used. One of the secondary case definitions will not exclude co-morbidities. In another secondary case definition, the presence of parasites at any density will be allowed. Because it is not clear what proportion of severe malaria cases will be in HIV-infected children and lack of previous experience on vaccine efficacy in HIV infected children, a third secondary case definition will exclude patients with a diagnosis of HIV infection (Table 1).

**Table 1:** Case definitions of severe malaria

Primary definition	Secondary definition 1	Secondary definition 2	Secondary definition 3
*P. falciparum *asexual parasitaemia >5,000 parasites/μL One or more markers of disease severity No co-morbidity	*P. falciparum *asexual parasitaemia >5,000 parasites/μL One or more markers of disease severity	*P. falciparum *asexual parasitaemia >0 parasites/μL One or more markers of disease severity No co-morbidity	*P. falciparum *asexual parasitaemia >5,000 parasites/μL One or more markers of disease severity No co-morbidity No HIV infection

Table 2 presents the clinical and laboratory markers that will be used to classify patients according to the case definitions. The clinical markers of disease severity are prostration, Blantyre coma score of two or less, two or more seizures and respiratory distress, characterized by lower chest wall in-drawing and abnormally deep breathing. The laboratory markers are hypoglycaemia, metabolic acidosis, lactic acidaemia and severe anaemia. Co-morbidities excluded from the primary case definition are pneumonia, meningitis, sepsis and gastroenteritis with severe dehydration.

**Table 2:** Markers of disease severity included in the severe malaria case definition

Marker of severity	Definition
Prostration	• Inability to perform a previously-acquired motor function:
	- Inability to stand in a child previously able to do so
	- Inability to sit in a child previously able to do so
	- Inability to suck in a very young child

Blantyre score ≤2	• Conscious level scored (0-5) using the Blantyre coma scale [27]. Determined by adding the scores for:
	- Best motor response (scored 0-2)
	- Best verbal response (scored 0-2)
	- Eye movement (scored 0-1)

Seizures, 2 or more	• Number of seizures occurring in the total time period including 24 h before admission, the emergency room and hospitalization

Respiratory distress	• Lower chest wall in-drawing or abnormally deep breathing

Hypoglycaemia	• Blood glucose <2.2 mmol/L

Metabolic acidosis	• Base excess ≤-10.0 mmol/L

Lactic acidaemia	• Lactate ≥5.0 mmol/L

Severe anaemia	• Haemoglobin <5.0 g/dL

## Markers of disease severity (Table 2)

Many studies have investigated the value of various clinical and laboratory markers as prognostic indicators of mortality and sequelae in malaria. In 1995, Marsh and colleagues showed that impaired consciousness, respiratory distress and severe anaemia were three central markers of disease severity associated with adverse outcome, especially when present together [19]. More recently, a two-year observational study of children in Gabon with *P. falciparum *malaria showed that coma, seizures, lactic acidaemia, respiratory distress and hypoglycaemia were independent predictors of death [20]. The highest fatality rate occurred in children with a combination of coma/seizures, lactic acidaemia and hypoglycaemia. A recent study of children in Mali showed that hypoglycaemia, respiratory distress and deep coma are independent predictors of death [21].

## Neurological impairments: prostration, Blantyre coma score, repeated seizures

Neurological impairment is a frequent marker of disease severity, and can result in long-term neurological sequelae [22]. In a retrospective study of patient records collected from 1992 to 2004 in a district hospital in Kenya, neurological involvement was detected in nearly half of 19,560 children with malaria [23]. The most common manifestations were seizures (37.5%), prostration (20.6%) and impaired consciousness or coma (13.2%). Numerous studies have demonstrated that coma/impaired consciousness and seizures are independently associated with mortality in African children with malaria [19-21,24-26]. In this phase III study, prostration, Blantyre coma score <2 and repeated seizures have been selected as markers of neurological impairment for the severe malaria case definition.

**Figure 1:**
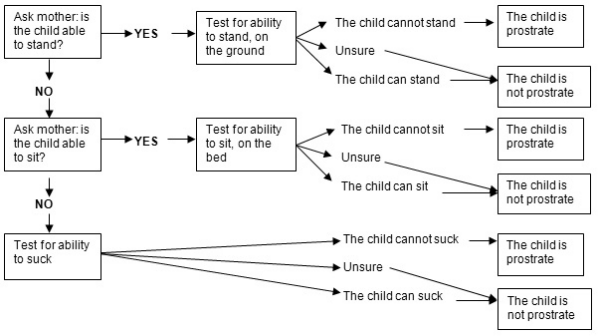
**Algorithm for evaluation of prostration**.

Prostration was included in the case definition of severe malaria as an important sign that will allow capture of many of the children that need hospital admission, surveillance, and prompt treatment to prevent further complications. Prostration is defined as the inability to perform a previously-acquired motor function, such as standing, sitting or, in very young children, the ability to suck, and is assessed in a child who is calm and awake. The trial algorithm for evaluating prostration is shown in Figure 1. Prostration is assessed at least one hour after control of seizures or hypoglycaemia.

**Figure 2:**
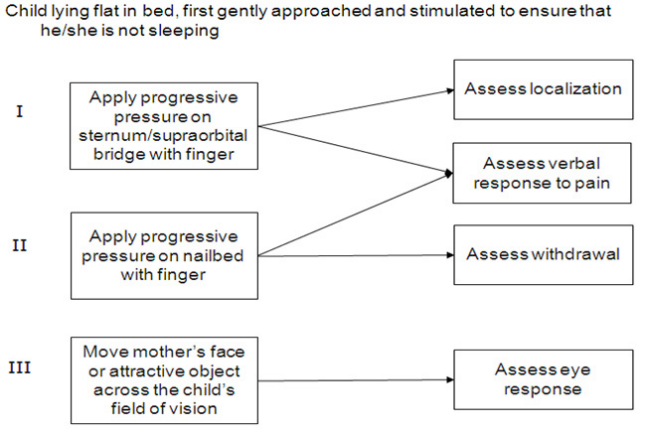
**The integrated Blantyre coma scale assessment**.

The Blantyre coma score provides a way of measuring conscious levels that is relatively easy to standardize across different sites. A low Blantyre coma score is associated with a high risk of an adverse outcome in malaria [6,27]. The score is based on motor, verbal and eye movement in response to stimuli. A score of 5 is normal, although under nine months of age a score of 4 can be normal, as the ability to localize a painful stimulus may not have been acquired by that age. A score of 2 or less is considered indicative of cerebral malaria [6,27]. The trial algorithm to evaluate Blantyre coma score is provided in Figure 2.

## Respiratory distress: deep breathing, lower chest wall in-drawing

Respiratory distress is also an important predictor of mortality in severe malaria [18-21,25,28-30]. Two specific markers of respiratory distress have been shown to be associated with an adverse outcome in malaria and are, therefore, included in the definition of severe malaria: abnormally deep breathing and lower chest wall in-drawing. Abnormally deep breathing is defined as deep or laboured respiration (acidotic or Kussmaul respiration) with increased inspiratory excursion and expiratory incursion of the chest. The respiratory rate may or may not be increased. Deep breathing is indicative of metabolic acidosis and associated with an increased risk of mortality in children with malaria [6,18,30]. The second marker of respiratory distress, lower chest wall in-drawing, is defined as the inward movement of the bony structure of the lower chest wall with inspiration. Chest wall in-drawing was independently associated with death in a study of 2400 malaria cases in Tanzania [27]. Other respiratory distress markers, such as nasal flaring or visible inward movement of intercostal tissues (intercostal recession), have not independently been shown to be associated with an adverse outcome in malaria [6].

## Metabolic complications; hypoglycaemia, acidosis, hyperlactacidaemia

Metabolic complications are common in severe malaria, particularly hypoglycaemia and acidosis [31]. Hypoglycaemia has been identified as a key prognostic indicator of high risk of death for children with malaria [6,18-20,23-25,27,32]. The risk of cognitive impairment following hypoglycaemia in malaria has also been described [21]. As no single clinical sign is a pathognomonic marker of hypoglycaemia, a blood measurement is required. In the phase III study, hypoglycaemia will be defined as a blood glucose concentration <2.2 mmol/L.

Acidosis, associated with lactic acidaemia and a low serum bicarbonate or base excess, has also been shown to be associated with an increased risk of mortality in patients with malaria [18,24,31,33-36]. The physiopathology of acidosis in malaria is complex. Studies have suggested key roles for both direct metabolic disturbances due to the parasitic infection, and a subsequent inflammatory response, and for a low circulatory blood volume [37,38]. The optimal cut-off points for lactic acidaemia and base excess that predict mortality have usually been around 5 mmol/L and from -8 to -12 mmol/L, respectively [32,39,40]. In the pivotal phase III study, acidosis will be defined by a negative base excess ≤-10.0 mmol/L or by lactic acidaemia ≥5.0 mmol/L.

## Severe anaemia

Severe malaria anaemia is a frequently overlooked contributor to overall malaria mortality, especially in young children. Studies have reported case fatality rates from severe malaria anaemia varying between 5.6% and 16%, with over 50% of deaths occurring within 24 hours of admission [18,27]. However, the exact mortality burden due to severe malarial anaemia is obscured by rapid transfusion in clinical research settings, which have enhanced case management practices that are unavailable to most children developing severe malaria anaemia [19,27,41]. Although anaemia is often multifactorial, the contribution of single or repeated malaria episodes is well recognized, and illustrated by the impact of malaria control measures on anaemia and by the peak in hospital admissions for severe anaemia and blood transfusion seen during the malaria season [6,42-44]. In the trial, severe anaemia, defined as a haemoglobin concentration <5.0 g/dL, has been included as one of the criteria of severe malaria.

## Markers of disease severity that were not selected for inclusion in the endpoint case definition

Markers of disease severity included in the case definition were chosen on the basis of their association with poor outcome, occurrence in a significant fraction of cases and an ability to standardize their measurement across sites. The selected markers also minimized data redundancy. For example, although circulatory collapse and delayed capillary refill time predicts mortality in children with severe malaria [39], it was agreed that patients with this indicator would be captured by the presence of prostration or acidosis. Markers of severity considered but not included were those that apply to only a small fraction of cases, are difficult to measure in a standardized way or are not part of accepted clinical practice or research methodology. For example, renal failure is not included as it is rarely seen in children with severe malaria [6]. Although retinal haemorrhage has been shown to correlate well with cerebral malaria [45,46], and is predictive of poor outcome [47,48], the method for monitoring retinal changes [49] would have been difficult to implement and standardize across sites.

## Exclusion from the severe malaria primary case definition upon presence of co-morbidities

While children in areas of intense malaria transmission may have *P. falciparum *parasitaemia and remain seemingly healthy, children admitted to hospital with *P. falciparum *parasitaemia often have an associated illness with symptoms which overlap with those of severe malaria [10,11,50,51]. Thus, parasitaemia in a severely ill child may sometimes be coincidental and not the cause of presenting symptoms. Four major, common infections have clinical features which can overlap with those of severe malaria: pneumonia, meningitis, sepsis and gastroenteritis with severe dehydration.

## Pneumonia

Several studies have demonstrated the difficulty of distinguishing between severe malaria and pneumonia, both of which are frequently associated with fever, tachypnoea and respiratory distress [10,52,53]. To capture cases of pneumonia in the phase III RTS, S/AS01 trial, a chest X-ray will be taken within 72 hours of admission in children with tachypnoea or respiratory distress. WHO guidelines on the interpretation of chest radiographs for the diagnosis of pneumonia in children will be employed [54], as illustrated in Table 3. Radiographically proven pneumonia will be defined as consolidation or pleural effusion (Table 4).

**Table 3:** Chest X-ray interpretation (WHO 2001)

Classification of quality of chest x-rays	
**Quality X-ray parameters**	**Classification of quality of chest x-rays (enter result in CRF)**

• ID on the right side • Position: clavicles and ribs symmetric on each side of the spine • Boundaries: rib cage and costophrenics angles seen • Inspiration: dome of the diaphragm is below the anterior tip of the 6th right rib • Movement: heart, diaphragm, central vessels and ribs sharply defined, without blurring • Exposure: vascular shadows can be seen in lung periphery, thoracic vertebrae and large lower lobe vessels visible through cardiac silhouette • Contrast: background outside patient's silhouette is black. Bones and airway easily distinguished from soft tissue	• **Uninterpretable: **if the features of the image are not interpretable without additional images. No further reading should be made for such images • **Suboptimal: **if the features allow interpretation of primary endpoint but not of other infiltrates or findings. No entries should be made for other infiltrates for such images • **Adequate: **if the features allow confident interpretation of endpoint as well as other infiltrates

**Classification of findings of chest x-rays**	

• **Consolidation or pleural effusion **:	
▪ where consolidation is defined as a dense opacity that may be a fluffy consolidation of a portion or whole of a lobe or of the entire lung, often containing air bronchograms*	
▪ where pleural effusion is defined if it occurs in the lateral pleural space (and not just in the minor or oblique fissure) and is spatially associated with a pulmonary parenchymal infiltrate (including other infiltrate) or if the effusion obliterates enough of the hemithorax to obscure an opacity	
* atelectasis of an entire lobe that produces a dense opacity and a positive silhouette sign with the mediastinal border will be considered to be an endpoint consolidation	
• **Other infiltrate **linear and patchy densities (interstitial infiltrate) in a lacy pattern involving both lungs, featuring peribronchial thickening and multiple areas of atelectasis. Lung inflation is normal to increased. It also includes minor patchy infiltrates that are not of sufficient magnitude to constitute primary end-point consolidation, and small areas of atelectasis which in children can be difficult to distinguish from consolidation.	
• **No consolidation, infiltrate or effusion**	

**Table 4:** Criteria for diagnosis of co-morbidities to be excluded from the primary case definition of severe malaria

Criteria for co-morbidity	Definition
Radiographically proven pneumonia	• Consolidation or pleural effusion on a chest X-ray taken within 72 h of admission

Meningitis on cerebrospinal fluid examination	• White cells ≥50 × 10^6^/L or positive culture of compatible organism or latex agglutination antigen detection test positive for Hib, pneumococci or meningococci

Sepsis (positive blood culture)	• Blood culture taken within 72 h of admission is considered positive if:
	- A definite pathogen is isolated (e.g. *Streptococcus pneumoniae*, *S agalactiae*, *S pyogenes*, *Haemophilus influenzae*, *Salmonella *spp.)
	- A bacteria that could be either a pathogen or a contaminant is isolated within 48 h of incubation (e.g. *Escherichia coli*, *Klebsiella pneumoniae*, *Staphylococcus aureus*, *Enterococcus faecalis*)

Gastroenteritis with dehydration	• A history of 3 or more loose or watery stools in previous 24 h and an observed watery stool with decreased skin turgor (>2 seconds for skin to return following skin pinch)

## Meningitis

Reliable clinical differentiation between severe malaria and meningitis can be difficult, impaired consciousness and seizures being cardinal symptoms of both [11,55]. Lumbar puncture is needed to confirm a diagnosis of meningitis, and to contribute to better treatment [56]. Establishing optimal criteria that indicate the need for a lumbar puncture has been the subject of considerable debate in all settings, and should be influenced by the background epidemiology of diseases affecting the central nervous system and meninges in the study area as well as by the availability of laboratory support. In malaria-endemic countries, the threshold for undertaking lumbar puncture should be low in order to differentiate between cerebral malaria and meningitis [11,57]. In the phase III RTS, S/AS01 trial, a lumbar puncture will be performed in the absence of contra-indications in children with an altered state of consciousness or seizures (excluding simple febrile seizures and seizures without fever in a known epileptic child). Patients with a cerebrospinal fluid (CSF) white blood cell (WBC) count ≥50 × 10^6^/L, a positive culture of an organism known to cause meningitis, or a positive Hib, pneumococcal or meningococcal latex agglutination antigen detection test will be excluded from the primary case definition of severe malaria (Table 4) [58]. A high CSF WBC count was selected as the threshold because cerebral malaria without meningitis can cause mild elevation of CSF WBC counts; patients with meningitis are likely to have WBC counts that are considerably higher than 50 × 10^6^/L [59]. This definition allows cases of cerebral malaria with only a mild increase in WBC count to be included in the primary analysis.

## Bacteraemia

Bacteraemia is common, and associated with a high mortality, in children admitted to hospital in sub-Saharan Africa [49]. Several studies have shown that invasive bacterial infections, clinically indistinguishable from severe malaria, are often under-diagnosed in malaria-endemic areas [50,60-62]. For this reason, all children in the phase III RTS, S/AS01 trial admitted to hospital for medical reasons (excluding planned admissions and surgical conditions) will have a blood culture taken on admission. Capture of cases of sepsis will be allowed up to 72 hours after admission (Table 4). Blood culture will be considered positive if a definite pathogen is isolated (for example, *Streptococcus pneumoniae*, *Streptococcus agalactiae*, *Streptococcus pyogenes*, *Haemophilus influenzae*, *Salmonella *spp.) or a bacteria that could be either a pathogen or a contaminant is isolated within 48 hours of incubation (for example, *Escherichia coli*, *Klebsiella pneumoniae*, *Staphylococcus aureus*, *Enterococcus faecalis*) [63].

## Gastroenteritis with severe dehydration

For the purposes of the trial, gastroenteritis with severe dehydration will be defined as a history of three or more loose or watery stools in the previous 24 hours and an observed watery stool with decreased skin turgor (>2 seconds for the skin to return to normal following a skin pinch) (Table 4). It was considered that this definition would allow the exclusion of cases of gastro-enteritis with dehydration severe enough to be the cause of prostration or acidosis but not cases of mild diarrhoea frequently associated with malaria. Decreased skin turgor is a key marker of severe intra-cellular dehydration, and so more typical of severe gastroenteritis than severe malaria. The exclusion of gastroenteritis cases with reduced skin turgor [64] will increase the specificity of the severe malaria endpoint [14].

Co-morbidities associated with malaria may not always be a coincidental, unrelated event. This is clearly demonstrated for invasive bacterial diseases, especially those due to Gram negative bacteria that can be a complication of severe malaria [65]. It will be important to know whether the malaria vaccine impacts on diseases other than malaria, through an indirect effect, as suggested by a previous phase II trial of RTS, S/AS01 [5] and by results from bed net trials [66]. Vaccine efficacy against severe malaria defined according to secondary case definitions, where patients with co-morbidities are not excluded from the analysis, will also be measured (Table 1).

## Implementation of standardized patient assessment

**Figure 3:**
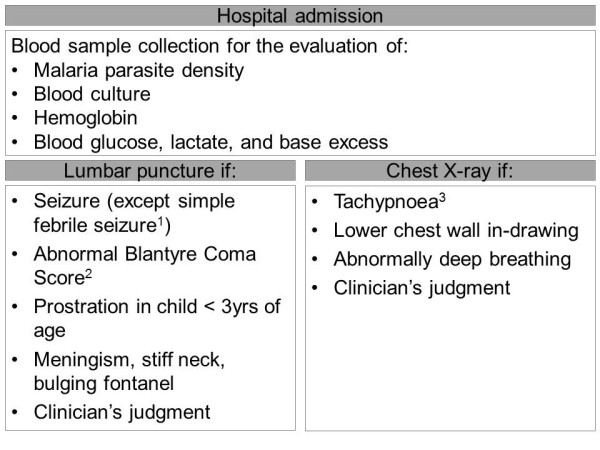
**Algorithm-based evaluation of severely sick children**. ^1 ^simple febrile seizure is defined as associated with fever, lasts for 5 minutes or less, generalized as opposed to focal, not followed by transient or persistent neurological abnormalities, occurring in a child ³ 6 months of age, with full recovery within 1 hour; ^2 ^BCS < 5 except in infants ≤ 9 months of age < 4, in association with best motor response of 1; ^3 ^≥ 50 breaths/minute in children under 1 year of age, and ≥ 40 breaths/minute in children over 1 year of age.

An algorithm for the evaluation of seriously sick children has been created to facilitate standardized identification and documentation of severe malaria and co-morbidities across research centres (Figure 3). This systematic assessment of hospital admissions will also promote the early diagnosis and treatment of these conditions.

The standardized management algorithm will require that for all acute hospital admissions (excluding trauma and planned admission), a blood sample is taken for the evaluation of haemoglobin, glucose, lactate, base excess, parasitaemia and blood culture, and that a case report form is completed. The decision to perform a lumbar puncture or chest X-ray will be made according to specific indications for each procedure and, in addition, if the clinician judges such an investigation likely to be useful to guide management. As far as possible, procedures and evaluations will be carried out in a child-friendly, relaxed environment, in the presence of a parent. Lumbar puncture will be indicated when children present with prostration, meningism (stiff neck or bulging fontanel), reduced conscious level (abnormal Blantyre coma score), or seizure other than a simple, generalized febrile seizure defined as one associated with fever, lasting ≤ 5 minutes, followed by no neurological abnormalities other than post-ictal sleep, and occurring in a child ≥ 6 months with full recovery within 1 hour. If there are signs of raised intracranial pressure, if there is no access to the puncture point or if lumbar puncture would compromise the child's respiratory efforts, then lumbar puncture will be delayed until after the patient's condition has stabilized. A chest X-ray will be done if there is tachypnoea or respiratory distress, especially lower chest wall in-drawing or abnormally deep breathing.

Implementation of standardized patient assessment is supported by a rigorous training and accreditation programme for all clinicians involved in patient care, including regular monitoring and evaluation during the trial. An online training and accreditation resource will be available to facilitate new staff training or refresher training of accredited staff if necessary.

Other provisions for optimizing general patient care, which were implemented in preparation of the trial, are summarized in Table 5. These include regular meetings of a Patient Care Forum, which gave lead clinicians from all centres the opportunity to discuss important areas of patient care, vaccine safety and reporting, clinical data collection and medical management of specific conditions. Copies of the WHO 'Pocket Book of Hospital Care for Children - Guidelines for the Management of Common Illnesses with Limited Resources' [67] were distributed and defined as a reference for hospital care, complementing local recommendations. Two doctors per research centre were trained in Paediatric Advanced Life Support. Access to recommended malaria control tools, such as impregnated bed nets and efficacious anti-malaria treatment is being facilitated.

**Table 5:** Provisions for optimising general patient care in the Phase III RTS, S/AS01_E _study

In study centres	In the field
• Paediatric care under the supervision of a qualified pediatrician	• Facilitate access to care and cover costs of care
• Clinical training and accreditation programme, with regular subsequent monitoring and evaluation	▪ Transport to hospital reimbursement
• Regular meetings of the Patient Care Forum to discuss patient care, vaccine safety, data collection, and management of specific conditions	▪ Local field workers to facilitate transportation
• Distribution of WHO Pocket Book of Hospital Care for Children	• Assure access to recommended prevention and treatment approaches:
• Improvements to care delivery that benefit the wider community	▪ Bed nets to be distributed during screening process in areas with no bed net access
• Improved infrastructure, wards refurbishment, introduction of new diagnostic techniques (microbiology, X-ray radiology)	▪ Where available, normal distribution channels encouraged through information sessions
• Hospital medical notes developed for patient follow up, extended to all admissions	▪ Exclude malaria treatments based on drugs with high resistance rates
• Address HIV/AIDS through collaboration with local HIV care delivery facilities	
• Local implementation of national recommendations access to voluntary counselling and testing	
• Access to highly active antiretroviral paediatric therapy at the local or regional level	
• Refer patients to hospitals with more comprehensive facilities when more precise diagnostic techniques or specialized care required	

The severe malaria case definitions described in this paper have been adopted for evaluation of vaccine efficacy against severe malaria at the trial analysis stage, and their use will not compromise the ability of study physicians to treat study children who present unwell with malaria, regardless of whether the criteria of standardized trial case definition are met, according to international guidelines and national recommendations. These are reviewed in one of the modules in the training and accreditation programme for investigators, covering aspects such as triage, nursing, emergency care, treatment of hypoglycaemia, convulsions, acidosis and anaemia (including precautions for blood transfusion), and use of antibiotics and anti-malarials.

The infrastructure of study centres has been improved to ensure that patients have standardized access to the assessment and diagnostic techniques needed for the study. This required patient access to care to be facilitated, wards to be refurbished and the introduction or strengthening of radiological and microbiological capacities (Table 4). These improvements to local facilities will remain beyond the trial and benefit the community at large. The clinical research know-how and infrastructures will reinforce the ability of research centres to diversify the research agenda, thereby reinforcing the sustainability of these improvements.

## Conclusion

Severe malaria has been identified by a WHO expert study group as an important endpoint for trials of malaria vaccines [8]. Its public health burden is sufficiently great to warrant its inclusion as a key endpoint and evidence of efficacy against severe malaria is likely to encourage implementation decisions when a vaccine becomes available. However, severe malaria is difficult to define, mainly because of the diversity in its clinical presentation and the frequent overlap of symptoms with other common illnesses. The definitions commonly used to diagnose severe malaria in clinical practice are not sufficiently specific for use in vaccine trials.

In the present study, the primary and secondary case definitions reflect the presentation of severe malaria that typically leads to hospitalization of young children in Africa. Furthermore, they are in accordance with the recommendations of the WHO [8], are locally applicable and appropriately balance sensitivity and specificity for the purpose of a clinical trial. A robust implementation programme ensures standardization of measurements across research centres. Furthermore, children will benefit from the diagnostic investigations introduced and the optimized clinical management of severe malaria cases. These strengthened capacities will benefit the local communities.

## Competing interests

JV and AL are employees of GSK Biologicals and hold stock options in GSK Biologicals. JV, KM, BG, AL, KPA, DA, JE, JS, PB, NS, PN, and SG declare their institution received a grant from PATH-MVI for the clinical trial described in this manuscript. JV and AL declare their institution has received grants from PATH-MVI for previous clinical trials. PB and PN declare their institution has grants pending from PATH-MVI. KM, BG, KPA, JE, JS, PB, NS, PN, MJH, SG, and DS declare receiving travel funds from PATH-MVI for travel related to this clinical trial. KPA declares his institution receiving a consultancy fee from PATH-MVI. JE and SG declare they or their institution receive financial compensation from PATH-MVI for administrative support. DS is employed by PATH-MVI. WK, SS, PK, and WO declare no potential conflicts of interest.

## Authors information

The following authors worked on behalf of the Clinical Trials Partnership Committee;

Kevin Marsh, Brian Greenwood, Amanda Leach, Kwaku Poku Asante, Daniel Ansong, Jennifer Evans, Jahit Sacarlal, Philip Bejon, Patricia Njuguna, Mary J Hamel and Samwel Gesase.

## Authors' contributions

All authors contributed to the development of this manuscript through discussions and document review. JV led the writing of the manuscript coordinating the incorporation of all reviewer comments. JV and DS coordinated the input of the PATH-MVI team. All authors critically contributed to the development of the case definitions of malaria and developed methods to standardize data collection through critical discussions and review of the protocol. KM, WK, SS, KPA, DA, JE, JS, PB, PK, NS, PN, MJH, WO, and SG contributed to the further development and implementation of the trial methodology at their research sites. All authors read and approved the final manuscript.
